# *BioSeqZip*: a collapser of NGS redundant reads for the optimization of sequence analysis

**DOI:** 10.1093/bioinformatics/btaa051

**Published:** 2020-01-30

**Authors:** Gianvito Urgese, Emanuele Parisi, Orazio Scicolone, Santa Di Cataldo, Elisa Ficarra

**Affiliations:** b1 Interuniversity Department of Regional and Urban Studies and Planning, Politecnico di Torino, Torino, Italy; b2 Department of Control and Computer Engineering, Politecnico di Torino, Torino, Italy

## Abstract

**Motivation:**

High-throughput next-generation sequencing can generate huge sequence files, whose analysis requires alignment algorithms that are typically very demanding in terms of memory and computational resources. This is a significant issue, especially for machines with limited hardware capabilities. As the redundancy of the sequences typically increases with coverage, collapsing such files into compact sets of non-redundant reads has the 2-fold advantage of reducing file size and speeding-up the alignment, avoiding to map the same sequence multiple times.

**Method:**

*BioSeqZip* generates compact and sorted lists of alignment-ready non-redundant sequences, keeping track of their occurrences in the raw files as well as of their quality score information. By exploiting a memory-constrained external sorting algorithm, it can be executed on either single- or multi-sample datasets even on computers with medium computational capabilities. On request, it can even re-expand the compacted files to their original state.

**Results:**

Our extensive experiments on RNA-Seq data show that *BioSeqZip* considerably brings down the computational costs of a standard sequence analysis pipeline, with particular benefits for the alignment procedures that typically have the highest requirements in terms of memory and execution time. In our tests, *BioSeqZip* was able to compact 2.7 billion of reads into 963 million of unique tags reducing the size of sequence files up to 70% and speeding-up the alignment by 50% at least.

**Availability and implementation:**

*BioSeqZip* is available at https://github.com/bioinformatics-polito/BioSeqZip.

**Supplementary information:**

Supplementary data are available at *Bioinformatics* online.

## 1 Introduction

In ultra-high-throughput sequencing, as many as 500 k sequencing-by-synthesis operations may be run in parallel, allowing the deep sequencing of DNA and RNA molecules in short time and producing a massive amount of data to be analyzed. With the ever-increasing production of new sequence data, and the continuous necessity of re-elaborating old data to extract hidden knowledge, new paradigms for data storage and analysis are becoming more and more critical (Muir *et al.*, 2016).

DNA and RNA reads are collected in the form of formatted files whose size can exceed a terabyte, typically storing two main types of information, both encoded by ASCII strings: (i) sequences, representing the bases of the biological molecules (RNA/DNA) and (ii) quality scores, representing the reliability level of each sequenced nucleotide.

The analysis of such RNA/DNA sequences starts with preliminary steps that consist of filtering the reads based on quality thresholds. Then, the high-quality reads are trimmed from the adapters and either aligned on a known reference database or assembled to construct new unknown genomes.

To have a more in-depth overview of the analysis flow, we can consider the example of RNA-Seq experiments. RNA-Seq reads, in either single-end (SE) or paired-end (PE) form, are first filtered based on their quality scores and trimmed from the adapters. Then, the most reliable reads (i.e. the ones with highest quality scores) are analyzed by applying one of three main strategies, as described by Conesa *et al.* (2016): (i) if a reference genome is available, reads are aligned to the genome with a gaped aligner. This approach allows the identification and quantification of known transcripts as well as the discovery of novel ones. (ii) If no novel transcript discovery is needed, reads can be mapped to the reference transcriptome by using an ungapped aligner. (iii) When no genome is available, reads are usually first assembled into transcripts. Then, reads are mapped back to the assembled reference transcriptome for quantification and annotation.

As far as the alignment problem is concerned, several tools are available (Mazzoni and Kadarmideen, 2016), mostly making use of suffix arrays to provide faster alignment compared to traditional Dynamic Programming methods (Smith and Waterman, 1981; Urgese *et al.*, 2014). Among the most representative: BWA (Li and Durbin, 2009), Bowtie2 (Langmead and Salzberg, 2012), STAR (Dobin *et al.*, 2013), Rail-RNA (Nellore *et al.*, 2017) and Yara (Siragusa, 2015). Even though the computational time of the alignment is greatly reduced compared to classic algorithms, it still remains prohibitive for most of the medium–low computational systems, like the work-stations that are commonly used in most bio-labs.

A possible solution to this problem is collapsing all the repeated reads into a single one, reducing the number of reads that need to be analyzed, and hence the size of the read file. The advantage of this solution is 2-fold: (i) the memory required for the storage can be reduced and (ii) more efficiently, the alignment algorithms can map each unique read to the reference only once, instead of multiple times.

To the best of our knowledge, there exist in the literature a few tools that already provide a collapsing option, but with several significant limitations. First of all, none of the tools to date allows controlling memory consumption. This is a critical lack, as it may make impossible to execute the collapsing programs on mid-low end platforms. Most of the tools are also limited in the type of data that can be processed. *SeqCluster (SC)* (Pantano *et al.*, 2011) can only collapse SE reads from small RNA-Seq datasets, while *FastUniq (FU)* (Xu *et al.*, 2012) can only deal with PE reads. However, the popular *FASTX-Toolkit* (*FXT*) (Gordon and Hannon, 2010) provides a simple function to collapse identical reads but does not keep any trace of the quality scores provided in the input files. On top of that, it cannot deal with PE samples. *Super* *Deduper* (*SD*) (Petersen *et al.*, 2015) is an interesting tool for PCR duplicate removal, as it examines only a small portion of each reads (called key) and automatically discards data containing unknown nucleotides (Ns). However, it is not able to solve the exact read collapsing problem. *ParDRe* (*PDR*) (González-Domínguez and Schmidt, 2016) has a reasonable run-time, but a significant memory consumption.

The full list of the literature tools, with collapsing functionality, is reported in Table 1, together with a summary of their respective features. In the order: the possibility to deal with SE and PE sequences, the way the quality score of the input sequences is processed (i.e. *AVG*: by averaging the quality scores of the redundant reads per nucleotide, *HIGH*: by retaining the best score per nucleotide, *SUM*: by summing up base qualities scores), the possibility to set a user-defined memory bound to the alignment, the supported file formats. As it can be easily gathered from the table, none of the tools provides a fully functional collapsing option, as they all lack some essential features.

**Table 1.** Features of the available collapsing tools, with our proposed tool *BioSeqZip* in the last row

Tool	SE	PE	Quality	Memory bound	File formats
*FU*	No	Yes	High	No	.fa.fq
*FXT*	Yes	No	No	No	.fa
*PDR*	Yes	Yes	High-AVG	No	.fa.fq
*SC*	Yes	No	AVG	No	.fa.fq
*SD*	Yes	Yes	High-sum	No	.fa.fq
*BSZ*	Yes	Yes	AVG	Yes	.fa.fq.sam.bam.tag.tagq

Collapsing procedures are widely adopted as a preliminary step for the alignment of sRNA-Seq on miRNA and other small non-coding RNA databases such as miRBase (Griffiths-Jones *et al.*, 2006), miRGeneDB (Fromm *et al.*, 2015,  2020) and piRBase (Zhang *et al.*, 2014). Tools designed to quantify miRNA expression levels such as *isomiR-SEA* (Urgese *et al.*, 2016), SeqBuster (Pantano *et al.*, 2010), sRNAbench (Barturen *et al.*, 2014) miRDeep2 (Friedländer *et al.*, 2012) and others (Desvignes *et al.*, 2019) take a simple file (defined as Tag file) as input, with the unique sequence in the first column and its number of occurrences in the second column, to minimize the number of calls to a computationally expensive alignment procedure. In our work, we leverage this concept and take it to a higher level.

In this paper, we propose *BioSeqZip*; a new approach to collapse redundant reads generated by next-generation sequencing (NGS) machines. The functionality of our proposed solution is 3-fold: (i) a read collapsing technique based on the external sorting algorithm allows limiting memory usage (Knuth, 1998). External sorting is required when the data being sorted do not fit into the RAM of a computing device and instead they must reside in the slower external memory (HDD or SSD). In our implementation, we use a hybrid sort-merge strategy. In the sorting phase, chunks of data small enough to fit in main memory are read, sorted and written out to a temporary file. In the merge phase, the sorted sub-files are combined into a single larger file. Thus, making the tool suitable not only to cluster computers, but even to medium systems with limited hardware capabilities. (ii) A multilevel collapsing procedure can be applied to compress even further the read files. Recurrent reads from different samples will be collapsed into a single file, where unique reads will occur only once. (iii) An integrated expansion procedure enables an easy restore of the read aligned files produced by mapping tools.

As it can be gathered from Table 1, our proposed solution addresses the lack of functionalities of the collapsing tools available to date (see the last row of the table). *BioSeqZip* generates compact files of unique reads storing the number of collapsed sequences, so that the number of detected molecules can be considered in the alignment algorithms, as well as their quality score information. On top of that, it supports seven different file formats, allowing easy integration to several classes of alignment, mapping and assembly algorithms.

To demonstrate the quality of our solutions, we tested the *BioSeqZip* compression module on 32 RNA-Seq samples from the Human BodyMap 2.0 dataset, collecting 2.34 billion of SE and PE reads. We assessed *BioSeqZip* performance in terms of run-time, memory consumption, reduction of reads number as well as of collapsed files size and compared our tool against five alternative tools in terms of both performance and usability. On top of that, we compared the performance of four different alignment algorithms on collapsed and raw/uncollapsed input files, respectively, with either SE or PE datasets.

## 2 Materials and methods

We propose *BioSeqZip* as a solution to the extensive computational time and memory requirements of NGS data analysis flows involving sequences, such as transcriptome/genome mapping and small RNA-Seq analysis. A common trait of these tasks is that they need to process all the reads of the sequenced samples multiple times before moving to the next steps of the analysis. Hence, these tasks are majorly benefited by the removal of redundant reads.

*BioSeqZip* is a read collapser that groups and counts the occurrence of the identical reads in the input sequence file, producing a minimal sorted list of unique reads (and the corresponding occurrence counts) ready for the alignment to a reference. The read collapsing can be obtained at two different levels: (i) at *single-sample* (SS) level, *BioSeqZip* collapses the redundant reads stored in a single file, producing a compacted output file with unique reads. (ii) At *multi-sample* (MS) level, *BioSeqZip* aggregates redundant reads from a dataset containing multiple sample files, allowing a supplementary level of aggregation.

In both cases, if the input files provide quality information about the reads, *BioSeqZip* returns the collapsed sequences with a quality score, that is computed as the average consensus quality of the corresponding collapsed reads. On top of that, *BioSeqZip* allows to choose the format of the collapsed file (either Fastq, Fasta, Tagq or Tagsee Supplementary Text S1 for the file format description). By doing so, the user can choose whether to maintain the quality information (and hence, bear the corresponding computational costs) or not, with a pay-for-what-you-use philosophy.

Besides the read collapsing functionality, *BioSeqZip* implements also a re-expanding functionality, that is able to recover the original occurrences of each sequence to update, with the number of read occurrences, the alignment files generated by mapping algorithms.

The functionalities of *BioSeqZip* are implemented into two main modules: a *Collapser* for compacting the redundant reads and an *Expander* for restoring aligned and compacted files. The modules were developed in C++ leveraging several packages of the SeqAn C++ bioinformatics library (Doring *et al.*, 2008; Reinert *et al.*, 2017). Most of our design choices were explicitly made to optimize the memory consumption and the computational performance on systems equipped with medium hardware resources. In the following, we describe the main design aspects, functionality and supported file formats of the *Collapser* and the *Expander* modules.

### 2.1 *BioSeqZip_Collapser* module

Collapsing the reads is a powerful strategy to reduce the computational time and complexity of the alignment steps of an RNA-Seq or DNA-Seq analysis pipeline. Indeed, in files with billion of reads, the same sequence will likely be analyzed multiple times. The collapser module collapses and counts the occurrence of redundant reads, that are detected based on their identical sequence. This new reduced collection of non-redundant sequences can be stored in a much smaller file and used for optimized alignment analysis. *BioSeqZip_Collapser* accepts as input Fasta, Fastq or SAM file formats and generates compact output with four different file formats: Fasta, Fastq, Tag and Tagq (Supplementary Text S1.1).

All the input/output file formats can be optionally provided in compressed form (gzip).

The most straightforward approach to collapsing reads is the one implemented by the *fastx_collapser* routine offered in *FASTX-Toolkit* (Giardine *et al.*, 2005). This solution requires to first load all the reads into the main memory, then to sort them, and finally to collapse them into unique tags, while counting and storing occurrence of the redundant reads. However, this approach is hugely memory-intensive, as it requires to load in memory the entire file that is typically in the order of tens of GBs.

Indeed, memory issues are always a critical aspect of NGS data analyses that typically exploit big data. As the dimension of the NGS files is generally huge, the amount of memory necessary to load a whole dataset will be overwhelming for medium–small memory systems such as standard workstations and laptops, leading to the necessity of leveraging powerful machines such as clusters of compute nodes and servers. Thus, increasing the costs of the analysis.

*BioSeqZip_Collapser* overcomes such limitation by implementing a memory-constrained collapsing functionality. For this purpose, the user is asked to set the maximum amount of the memory that can be exploited by the program, based on the capabilities of the available hardware.

**Fig. 1.**
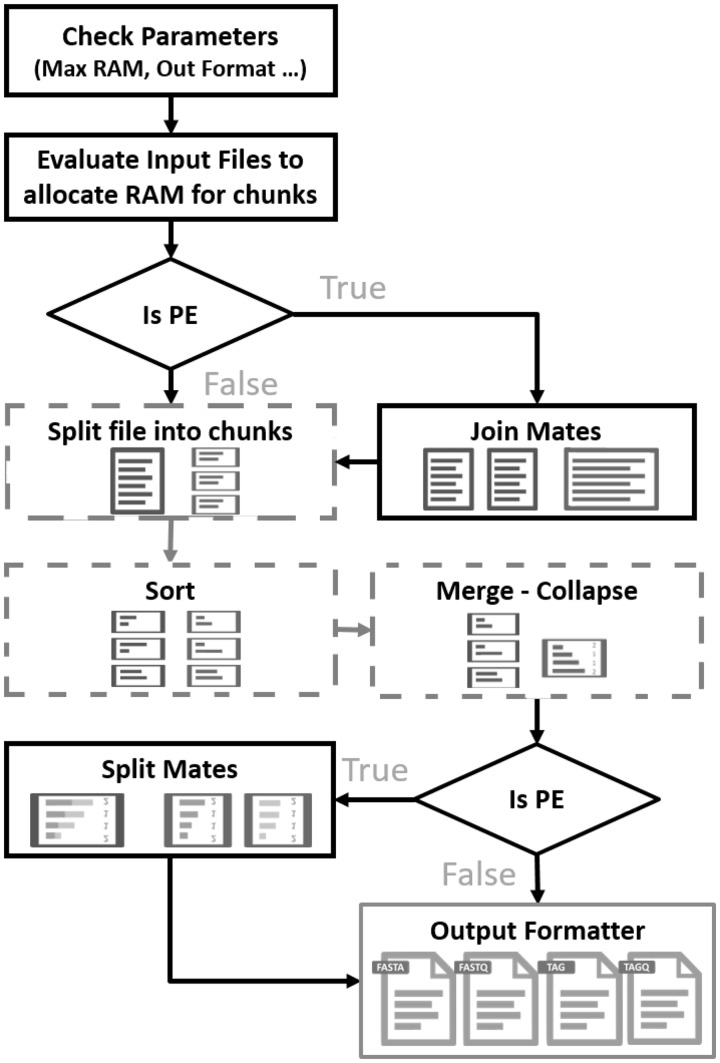
*BioSeqZip*_Collapser flowchart, with external sorting and collapsing steps highlighted in dashed boxes

The main steps of *BioSeqZip_Collapser* are shown in Figure 1.

In the first phase, we pass and check the input parameters: *–input* and *–max-ram* respectively for the input file storing the reads to be collapsed (either Fasta, Fastq, SAM or BAM) and the maximum amount of RAM allowed. A set of reads (*T*_size_) are read from the input file to evaluate their memory occupancy (*T*_RAM_). Then, we estimate the maximum number of reads (*C*_size_) that can be loaded at the same time into memory without exceeding the memory constraint, set by the user (*C*_RAM_), by computing the Equation (1) where *α* is an empirical correction factor.

(1) $$C_{\text{size}}=\alpha \cdot \frac{C_{\text{RAM}}\cdot T_{\text{size}}}{T_{\text{RAM}}}.$$

**Fig. 2.**
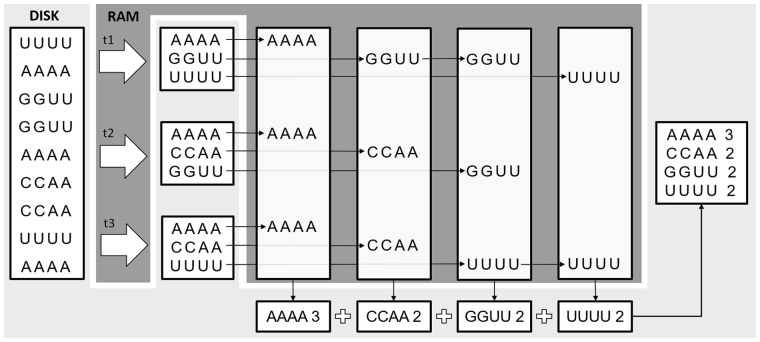
Example of read collapsing based on external sorting

The module implements a custom *parallel external sorting* procedure (Knuth, 1998) that sorts the reads in chunks and generates *M* temporary files of sorted records. For clarity, in Figure 2 we show a schematic example of the External-Sort-Collapsing procedure. Each chunk of reads (of size 3, in the example of Fig. 2) is loaded into RAM, where it is alphabetically sorted by a multi-threaded function. Then, the sorted set of reads are collapsed and written on a temporary file on the disk (in dark-gray background). In the  phase, the first $C_{\text{size}}/M$ sequences from all the temporary files are loaded in *M* buffers. Then, an iterative selection-sort process is started, for identifying the overall occurrences count of each read. At each iteration, the tool appends a collapsed sequence to the output file, together with the corresponding occurrences count. Empty chunks are refilled with a portion of the temporary file from which the collapsed reads come. At the end of the merge–collapse procedure, the output file will contain all the collapsed reads and their corresponding occurrence count. If requested by the user, in case the input file provided a quality score per read, the procedure computes the average quality score of each block of collapsed reads, base-by-base.

Besides the aforementioned, *BioSeqZip_Collapser* supports two additional cases: (i) files with PE reads stored with the interleaved format, where mate 1 is in the even reads and mate 2 in the odd reads. (ii) Files with joined mates and fixed break-point, where the two mates are already in the same read.

As far as file formats are concerned, six different input/output combinations are available, all optimized to minimize the number of required operations. More specifically, with Fastq input, the user can select any of the four output formats. With a Fasta format, however, the output can be either Tag or Fasta. Optionally, *BioSeqZip_Collapser* can be even forced to generate a collection of output files with constant size set by the user. In this case, the module generates multiple files of the set dimension, each storing a portion of the collapsed reads sorted in alphabetical order. This feature is particularly useful in cases where the read files must be distributed to an High Performance Computing  cluster. For Fasta and Fastq output formats, the read count is appended to the identifier on the header line. By doing so, the output file can be easily handled by alignment programs without losing track of the original number of redundant reads before collapsing.

If the input is not a single file, but a folder containing multiple files, the *BioSeqZip_Collapser* will implement a MS collapsing strategy. Even in this case, the procedure can generate any of the four possible output files, with a supplementary table reporting the ID of collapsed sequence in the first column, the sequence in the second column and its per-sample occurrence counts in the following columns. The information reported in this table can be eventually leveraged by the *Expander* module to recover the original occurrence of redundant reads in each sample.

### 2.2 *BioSeqZip_Expander* module

The files storing the collapsed reads can be given as input to the most common RNA-Seq and DNA-Seq analysis pipelines, thereby considerably reducing demands in terms of storage space and computational time. As already discussed, this is a significant advantage in case of systems with limited hardware resources that might not be powerful enough to host the analysis of redundant data.

Especially for mapping and alignment procedures, the best practice would be to consider the complete available information at the same time (i.e. sequence, sequence occurrence count and average quality score). However, standard alignment tools are generally unable to leverage the occurrence count field of Fasta/Fastq files. Hence, the analyst is left with two options: (i) customizing the mapping procedure to consider the read counts during the alignment algorithm [We have adapted BWA and Yara aligners to consider the occurrences stored in the header of each read of the collapsed Fastq files, and the modified code is provided in two additional GitHub repositories (links provided in the Supplementary Availability section)]. (ii) Align the not-redundant reads and then re-expand the compact aligned files, exploiting the occurrence counts to replicate the original entries. The latter functionality is implemented by *BioSeqZip_Expander*. This module re-expands the output files of alignment/mapping tools by replicating each Tag by the occurrence count of each sample. By doing so, it restores an expanded collection of redundant sequences and corresponding mapping locations.

Multi-mapped reads can be handled either with a random assignation strategy, as done by BWA (Li and Durbin, 2009), or with a first position policy, like Yara (Siragusa, 2015), where the additional locus are listed in a custom field of the SAM file.

*BioSeqZip_Expander* is meant for re-expanding the output of any alignment tool, to obtain the same output that would have been generated by aligning the raw non-collapsed sample. As long as each SAM record stores the original name of the aligned read, *BioSeqZip_Expander* is able to parse it and extract the number of times the sequence was found by the *BioSeqZip_Collapser* in the raw sample. Then, the target SAM record is written that number of time to the final output file, which represents the expanded alignment file. *BioSeqZip_Expander* can deal with the two most common file formats for alignment files: SAM and BAM.

In the case of MS collapsing, *BioSeqZip_Expander* will exploit the supplementary table generated by the MS collapsing strategy to restore the correct number of read sequence and originally detected in each corresponding sample.

## 3 Results and discussion

We tested *BioSeqZip* on a set of 32 transcriptomic samples of 16 different human tissues from the Human Body Map 2.0 Project (ERP000546). This set, characterized by the presence of both SE and PE reads, was generated with the HiSeq 2000 sequencing technology (Ogasawara *et al.*, 2006).

To assess our methodology, we evaluated four popular alignment tools on both collapsed and not collapsed reads. We decided to evaluate the quality and computational costs of the alignment, because it is the fundamental step of any sequencing data analysis.

The experiments were performed on a Linux machine with 2 × 8 Intel cores clocked at 2.4 GHz (Xeon E5-2630), 128 GB RAM, 16 TB HDD SAS in RAID 6.

In the following, we will show the performance of our read collapsing algorithm and the benefits of using tags representing collapsed sequences for mapping procedures. We collapsed Fastq files, hence retaining the average quality of the original files. A quality-agnostic compression would be twice as faster and produce collapsed files of half the size of the case reported here. Thus, the time and memory reported in our results should be considered as a worst-case scenario.

### 3.1 SS and MS collapsing performance

In this experiment, we collapsed reads from 16 SE and 16 PE human samples (see full list and details in Supplementary Table S1). SE samples containing 1.26 billion of 75 bp reads, while PE samples comprising 1.28 billion of 50*2 bp Illumina reads. Both classes of samples sequenced with a 20× coverage at a genome size of 3.1 GB. Each sample contains 80 million of reads on average and occupies 17 GB on the disk for the SE and 26 GB for the PE.

In our test, the raw files were collapsed using *BioSeqZip_Collapser*, first with a SS and then with a MS strategy. Table 2 shows the results of the collapsing procedure, in terms of the number of reads, the total size of samples and run-time. The first column of the table makes explicit whether the considered samples are uncompressed (i.e. RAW), collapsed SS or collapsed MS. The second column indicates whether the samples are SE or PE reads. The following two columns report the number of reads and file size. Concerning RAW and SSs, the sum of the corresponding sizes for each sample is reported. While for MSs, the results were produced by the MS collapse procedure. Finally, the last three columns of the table report the overall collapsing time and the gain [computed with Equation (2)] of the collapsing, in terms of number of reads to be aligned and size of the file on the disk.

(2) $${\text{Gain}}_{\%}=\left(1-\frac{\text{Collapsed}}{\text{Raw}}\right)\cdot 100.$$

**Table 2.** SS and MS collapsing performance

Sample		No. of reads (G)	Size (GB)	Collapse time (s)	Gain	
Status	Type				No. of reads (%)	Size (%)
RAW	PE	1.28	390	0	0	0
	SE	1.26	252	0	0	0
SS	PE	0.59	141	9633	53.7	63.8
	SE	0.39	64	6106	69.4	74.6
MS	PE	0.54	134	12 708	57.7	65.6
	SE	0.28	47	7763	78.0	81.4

Some interesting observations can be gathered from Table 2. First of all, SE samples achieved higher collapsing rate than PE samples. This is due to the exact collapsing algorithm leveraged by *BioSeqZip*, that has better performance for shorter sequences, assuming similar datasets sizes. More specifically, collapsing all the SE samples with a SS strategy leads to a reduction in the number of reads to be analyzed of ∼70%, whereas the storage requirements are reduced to a quarter. MS collapsing leads to an even better gain in terms of the number of reads and files size, but it comes at the cost of higher run-time (30% higher in the worst case). In Supplementary Section S4.2, we propose a collapsing experiment on a WGS DNA-Seq sample from Scherer *et al*. (2019), showing the benefit of the collapsing strategy on this type of data.

Different values of the memory constraints translate into a different number of intermediate files that are generated during the collapsing (the lower the memory limit, the higher the number of disk operations). To clarify the relation between memory limit and performance, we run an additional experiment by collapsing three random files, at 4, 8, 16 and 32 GB limits by using 4 threads. The run-time in seconds was respectively 1062, 1098, 1172 and 840. This analysis highlights that the run-time required for collapsing a sample increases as the size of the buffer used for storing the sequences increases, up to the amount of memory that can contain the full file to be collapsed (more details in Supplementary Section S4.3).

### 3.2 Alignment performance on collapsed samples

To assess the impact on alignment performance of both SS and MS collapsing, we proceeded as follows: (i) we performed SS collapsing of all the samples. Then, each collapsed file was aligned to the Human transcriptome (Cunningham *et al.*, 2015) using four popular read mappers: respectively, BWA, Bowtie2, Yara and STAR. Alongside, the raw uncollapsed files were also aligned using the same tools, to measure the alignment time on the original number of reads. (ii) We performed MS collapsing of the whole dataset, measuring both execution time and RAM requirements (always within the input constraint). Thus, we obtained a single collapsed Fastq output file and a table of read occurrences for the whole dataset. Then, we run the mappers on the Fastq file, assessing the execution time of the alignment as well as the storage size of the mapped reads.

In both experiments, we did not run the Expander module, which is conceived as an optional feature in regular downstream analysis.

**Fig. 3.**
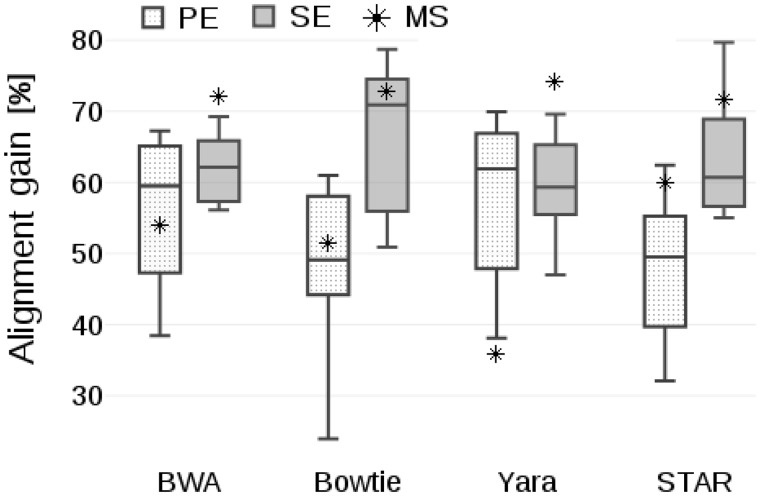
Gain of aligning collapsed files, using alignment time as the figure of merit

The obtained results are plotted in Figure 3, where we show the gain of aligning collapsed files (either SS or MS) instead of raw ones, using alignment time as the figure of merit in Equation (2). For the SS files, the collective gains of the individual samples of the dataset are reported in the form of a box-plot, while an asterisk represents the gain of MS. As can be gathered from the plots of Figure 3, the benefits of collapsing were consistently high for all the mapping tools. More specifically, the average alignment time speed-up attested between 50% and 70% in case of SS collapsing and between 36% and 73% for MS collapsing.

If we take into account the storage requirements, the impact of collapsing was even more significant, as the mapping file size was reduced by 71% with SS and up to 80% with MS collapsing, respectively. On top of that, we registered a significant reduction in the size of the collapsed alignment files. Indeed, for the overall alignment files the total disk occupancy of raw files aligned was 4.7 TB while SS aligned files occupy 1.9 TB with a reduction of 60%. However, the best size reduction was achieved when aligning MS collapsed files, obtaining a disk space reduction of 66%.

**Fig. 4.**
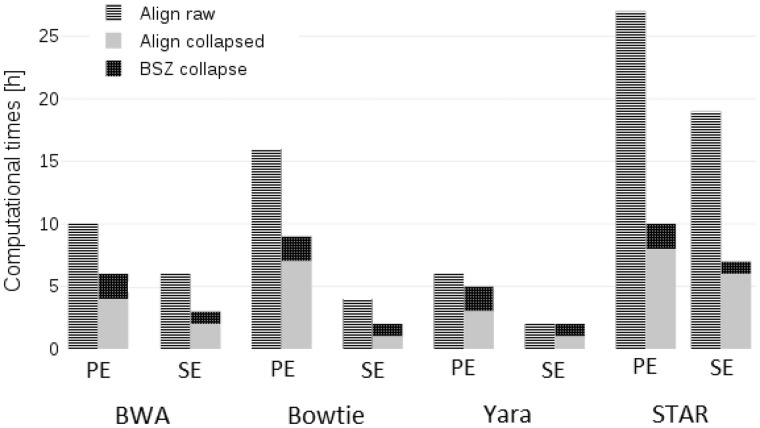
Alignment performance on raw and collapsed files

To estimate the run-time benefits of using collapsed files, in Figure 4 we show the results obtained by aligning the raw SE and PE files alongside the SS collapsed. In this plot, to have a complete view, we took into account even the additional computational time needed by *BioSeqZip* to collapse the sequence files (blue portion of the bars). As it can be gathered from Figure 4, applying *BioSeqZip* had a significant impact on the computational time required by alignment and mapping procedures. The highest impact was for the tool STAR, for which time reduced from 19 to 7 h for SE and from 27 to 10 h for PE, with reductions attesting between 55% and 63%, respectively. Collapsing was very advantageous also for BWA and Bowtie2. The weakest impact was for Yara mapper, for which the alignment time of samples of the size considered in our tests is already minimal. Nonetheless, as it is visible from the blue portion of the stacked bars, the additional computational time required for collapsing the sequence file is always a small fraction of the overall time needed to execute the alignment on the raw reads (red bars). Hence, the collapsing is advantageous even in the worst-case scenario.

To avoid possible inconsistencies, in our tests we verified the coherence of the raw and collapsed alignment files, by checking that the same number of reads where mapped by the tools to the same genome location of the alignments produced using the raw Fastq files.

In the last mapping experiment, we analyzed SE samples of BodyMap with either BioSeqZip_Collapser + STAR + BioSeqZip_Expander (BSZ_C+STAR+BSZ_E) and Rail-RNA alone and compared the two runs in terms of both execution time and memory consumption (full analysis in Supplementary Section S4.4). From this experiment, we obtained that the combination of BSZ_C+STAR+BSZ_E outperformed Rail-RNA by 68% (15.29 versus 47.37 h). Note that the comparison is limited to time and memory performance of the two pipelines, without in-depth evaluation of the accuracy and consistency of the provided output.

### 3.3 Comparison with alternative collapsers

For this test, we randomly selected three SE and three PE samples (listed with blue background in Supplementary Table S3) and used them to compare the compression performance achieved by *BioSeqZip* with other tools available in the literature. The SE samples (labeled E890, E894 and E902) contain respectively 64, 77 and 82 million of 75 bp reads, with a disk space occupancy of ∼19, 22 and 24 GB. The PE samples (labeled E873, E882 and E886) are characterized by 82, 74 and 83 million of 50*2 bp reads, with a disk space occupancy of 25, 22 and 25 GB.

In our test, the raw files were collapsed using *BioSeqZip_Collapser (BSZ)*, *Fast Unique (FU)*, *FASTX-Toolkit (FXT)*, *ParDRe (PD)*, *SeqClaster (SC)* and *Super Deduper (SD)*, respectively. In case a tool did not support either PE (e.g. *FXT* and *SC*) or SE (e.g. *FU*), we implemented a specific adapter to convert SE into PE and *vice* *versa*. Indeed, some of the tools considered in our test match the collapser requirements only partially, because they were not specifically designed for this task (see Table 1). All the tools were executed with default parameters, killing the process if running for more than 5000 s.

**Fig. 5.**
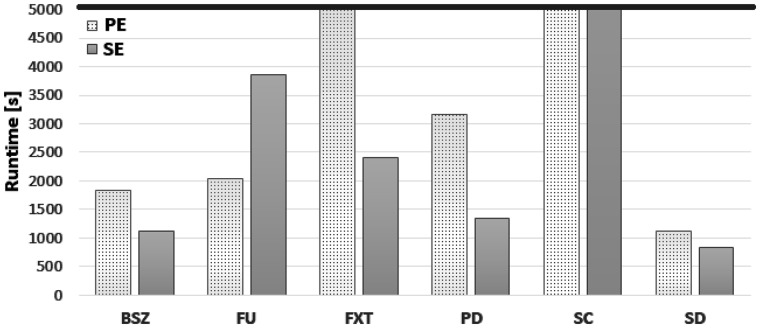
Collapsing performance of different collapsing tools. Total computational time for collapsing SE and PE samples, respectively

Figure 5 reports six groups of bars, one per tool, showing the performance in terms of total computational time spent for collapsing the three SE and the three PE samples, respectively. As it can be observed from Figure 5, *BioSeqZip* was faster than all the other tools except for *Super Deduper*. However, *Super Deduper* collapses the reads by only looking at the first bases of the sequence. Thus, it generates over-collapsed files where reads with a different sequence are often grouped together (more details in Supplementary Fig. S1, where the collapsing efficiency of each tool is duly reported). *FASTX* was close to our tool when dealing with SE samples, but on the other hand it completely overlooks quality information and generates only Fasta files as output format. Similarly, *ParDRe* obtained a performance comparable to *BioSeqZip* on PE samples, but even in this case not all the identical reads were detected and collapsed (details in Supplementary Fig. S1).

**Fig. 6.**
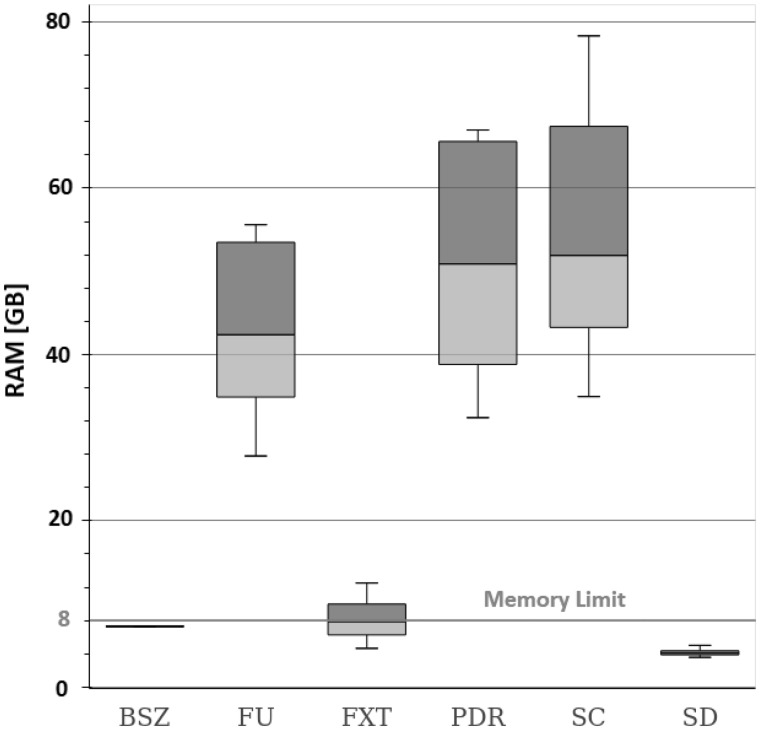
Memory usage of collapser tools

As already pointed out, a major distinguishing point of our implementation is the possibility to impose a maximum limit of memory that can never be exceeded. In Figure 6, we report the memory consumption of *BioSeqZip* with an imposed memory limit of 8 GB and show the memory consumption of all the other tools for comparison.The amount of RAM used impacts on the speed of *BioSeqZip*. The more RAM we use, the lesser reading/writing operations we perform on the disk. Since the disk operations are the bottleneck of our algorithm, reducing the number of these operations has a positive impact on the overall run-time of the *BioSeqZip* collapsing procedure. As can be gathered from Figure 6, three tools out of six reached peeks of 78 GB when dealing with PE samples. Only *FXT* and *SD* were close to the 8 GB limit, even though, unlike our solution, there is no specific guarantee that this constraint is always respected. In our tests, *FXT* tool reached a peak of RAM consumption close to 13 GB, but without keeping tracks of quality information. As already discussed, *Super Deduper* obtained the best performance only in theory, as it over-collapsed non-identical reads.

In summary, *BioSeqZip* provides the best advantages in terms of run-time and memory usage. Moreover, it generates alphabetically sorted files, which is very useful to reduce the complexity of downstream analysis.

### 3.4 Overall impact of *BioSeqZip* on alignment

In this last section, we aggregate the outcome of the previous tests to estimate the overall advantages of *BioSeqZip* in terms of reduction of the file size on disk and of computational time spent for the mapping.

As far as disk usage is concerned, the overall size of the collapsed files, plus mapped files obtained using collapsed files as input, was 2.1 TB for SS and 1.8 TB for MS, against 5.4 TB of the RAW files. More specifically, *BioSeqZip* was able to reduce 2.54 billion of reads into 980 million of unique tags, in <4.4 h of computations.

In Table 3, we show details about alignment time of BWA, Bowtie2, Yara and STAR on raw and SS collapsed reads, respectively. The last column of the table reports the gain, using the execution time of the alignment procedures on raw and SS collapsed files as the figure of merit in Equation (2). As it can be gathered from the table, when using Yara on collapsed files we observed a gain of 48%. The alignment time reduction was even more significant for the other mapping tools: 57% for BWA, 55% for Bowtie2 and 68% for STAR.

**Table 3.** Overall impact of *BioSeqZip* on the alignment time

Mapper tool	Alignment time (h)		
	Raw read file	Collapsed tag file	Gain (%)
Yara	9.1	4.7	48
BWA	16.3	7.05	57
Bowtie2	21.2	9.5	55
STAR	47.2	15.2	68

Based on the values of Table 3, we can estimate a total saved time equal to 57.3 h, which provides the overall figure of merit of the impact of *BioSeqZip* on alignment time.

## 4 Conclusion

In this paper, we presented *BioSeqZip*, a read collapsing tool that can reduce the huge sequence files generated by high-throughput NGS machines to compact sets of non-redundant reads.

As we extensively demonstrated in our experiments, *BioSeqZip* brings down the computational requirements of sequence analysis pipelines, with particular benefits for the alignment techniques, that are typically the ones with the highest computational costs.

In summary, the virtues of our approach are manifold:

- it is hardware-adaptive, as it is able to constrain RAM utilization based on a user-defined threshold depending on the hardware capabilities of the system.
- it is flexible, as it supports all the main sequencing file formats, and operates at either an SS or an MS level.
- it is exhaustive, as it maintains track of read quality information and read occurrence counts while collapsing the redundant sequences, allowing easy restoration of the original data.

Besides alignment, we firmly believe that the complete analysis pipeline will significantly benefit from the application of our collapsing strategy. With minor modifications, mapping and alignment tools may even directly leverage the occurrence counts information provided by the compressed files to skip the re-expansion phase and speed-up the mapping process (BWA and Yara are already available). Exploiting sorting and merging operations, performed by *BioSeqZip* during the collapsing phase, the mapped files (in SAM/BAM formats) will be ready for further analysis as-is, without needing any supplementary manipulations. Thus, in perspective, the computational costs of the overall analysis can be reduced even further.

Sure indeed, the collapsing provided by *BioSeqZip* comes at a possible cost of losing the read-specific quality. However, in case of need the original quality value can be easily recovered from the full uncollapsed Fastq.

## Supplementary Material

btaa051_Supplementary_Data
