# Supplementary material for: BioSeqZip: a collapser of NGS redundant reads for the optimization of sequence analysis
Source: Bioinformatics. 2020 Jan 30;36(9):2705–11. doi: 10.1093/bioinformatics/btaa051 (PMC7203750; doi:10.1093/bioinformatics/btaa051)
Supplement: btaa051_Supplementary_Data [file btaa051_supplementary_data.pdf]

SUPPLEMENTARY MATERIAL OF BIOSEQZIP: A COLLAPSER OF NGS REDUNDANT READS FOR THE OPTIMISATION OF SEQUENCE ANALYSIS

A PREPRINT

Gianvito Urgese
Interuniversity Department of Regional and Urban Studies and Planning
Politecnico di Torino
Torino, Italy 10129
gianvito.urgese@polito.it

Emanuele Parisi
Department of Control and Computer Engineering
Politecnico di Torino
Torino, Italy 10129
emanuele.parisi@polito.it

Orazio Scicolone
Department of Control and Computer Engineering
Politecnico di Torino
Torino, Italy 10129
orazio.scicolone@polito.it

Santa Di Cataldo
Department of Control and Computer Engineering
Politecnico di Torino
Torino, Italy 10129
santa.dicataldo@polito.it

Elisa Ficarra
Department of Control and Computer Engineering
Politecnico di Torino
Torino, Italy 10129
elisa.ficarra@polito.it

December 2, 2019

ABSTRACT

In this document the supplementary material of the paper *BioSeqZip: a collapser of NGS redundant reads for the optimisation of sequence analysis*

***K*eywords** Read collapser · NGS · RNA-Seq · Sequence aligner · Mapper · DNA-Seq · External sort

1 Supplementary Text

1.1 In/Out File Formats of *BioSeqZip*_Collapser

BioSeqZip_Collapser accepts as input Fasta, Fastq, Sam, and Bam file formats and generates compact output with four different file formats: Fasta, Fastq, Tag, and Tagq, respectively. All the input/output file formats can be optionally provided in compressed form (gzip). In the following we provide a brief summary of the key features of these formats.

- *FASTA* is a text-based file format used to represent either nucleotide or amino acids sequences. A sequence begins with a single-line description, followed by lines containing the sequence data. The description line begins with the sequence ID and is characterised by a '>' (greater than) symbol as the first character of the string. Following lines contain strings of characters representing the bio-molecule, using an appropriate alphabet for the encoding (*ACGTN* for DNA and *ACGUN* for RNA). Each sequence ends when another line starts with a '>' symbol.
- *FASTQ* format embeds in a FASTA-like file additional information about the quality with which sequences were produced by the NGS machines. The quality scores are encoded with a single ASCII character: the '!'

character represents the lowest quality and the '∼' character the highest, respectively. In a FASTQ file each sequence is represented on four lines: i) the first line start with a '@' character and is followed by a sequence identifier and an optional description (like in a FASTA title line). ii) the second line contains the sequence of letters of a read. iii) the third line begins with a '+' character; iv) the fourth line encodes the quality values of the sequence, one per base. Hence, it must contain the same number of symbols as line two.

- *Tag* and *Tagq* file formats are customized output files of *BioSeqZip_Collapser*, and have a column-based structure that is usually adopted by the smallRNA-Seq analysis tools. Tagq files have three columns: i) in the first column, the unique sequence (called tag) obtained after the read collapsing. ii) in the second column, the tag quality, obtained as the average quality of the collapsed sequences. iii) in the third column, the number of identical sequences in the input file that were collapsed to generate the tag. Tag files will have the same structure but with two columns only, as the quality information is not provided in this format.

1.2 In/Out File Formats of *BioSeqZip*_Expander

BioSeqZip_Expander can work on SAM and BAM file formats detailed in the following.

- *SAM* format is used for storing sequence data, either aligned or unaligned, in a human readable format [?]. A header section provides a list of reference sequences as well as other supplementary information provided by the alignment tool. In the second part, the file lists all the reads and the mapping positions on the reference in a tabular format. Each line includes eleven mandatory fields, the most important ones being the query name, the reference name, the position in the reference, and the read sequence).
- *BAM* format provides a binary version of the same data available in the SAM format. Clear advantage of this format is that it can be efficiently compressed to reduce storage occupancy.

2 Supplementary Availability

For the *BioSeqZip* project, we provide the following three repositories:

- https://github.com/bioinformatics-polito/BioSeqZip.git containing the *BioSeqZip*source code implementing the collapser and expander functionalities.
- https://github.com/bioinformatics-polito/BioSeqZip-BWA.git containing a modified version of BWA capable of using as input the read files generated by *BioSeqZip* for creating the full mapping files. This tool version does not need an expansion procedure of the output mapping files.
- https://github.com/bioinformatics-polito/BioSeqZip-Yara.git containing a modified version of Yara capable of using as input the read files generated by *BioSeqZip* for creating the full mapping files. This tool version does not need an expansion procedure of the output mapping files.

3 Supplementary Figures

	S1	S2	S3	S4	S5	S6
BSZ	49.2%	71.8%	63.0%	47.0%	27.3%	53.6%
FU	49.2%	71.8%	63.0%	47.0%	27.3%	53.6%
FXT	49.2%	71.8%	63.0%	47.0%	27.3%	53.6%
PDR	49.2%	71.8%	63.0%	45.4%	26.4%	52.1%
SC	49.2%	71.8%	63.0%	47.0%	27.3%	53.6%
SD	71.3%	77.1%	73.2%	62.9%	65.4%	66.7%

Figure 1: Experimental comparison of collapsed files generated with different collapsing tools. In green, instances where the output file contained only non-redundant reads. In yellow, instances where not all the redundant reads were collapsed. In red, instances where the input files were over-collapsed (i.e. not equal reads are grouped together) and the coherent representation of the read set is lost.

4 Supplementary Analysis

4.1 Collapsing performance on Single Cell samples

For this experiment, we used the two Single-Cell RNA-Seq samples from 10-week human embryo forebrain tissues (SRP129388), characterized by a conspicuous number of reads: in total 505 million reads with a 98 bp length. These samples were sequenced by Illumina HiSeq 2500 sequencing technology and the original files are 150GB large.

The results of this test are shown in the barchart of *Figure 2*. More specifically, the five groups of bars in the figure show the impact of the tool on the analysis of two samples (S906 and S907), in terms of % reduction of five figures of merit: file size, number of reads, computational time for mapping with Yara, Bowtie2 and BWA, respectively.

The same alignment time reductions shown in this chart were also experienced when using as a reference the Human Genome HG38.

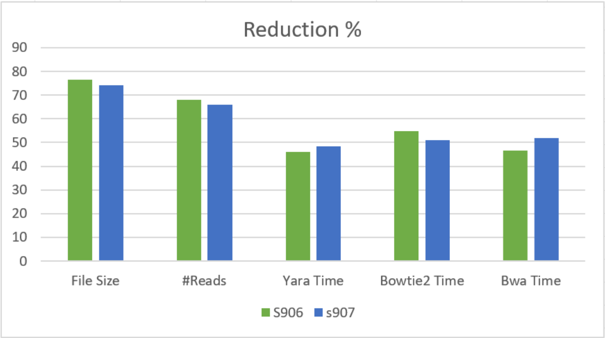

Figure 2: Collapsing performance on Single Cell samples. Bars represent the percentage reduction in terms of file size, number of reads, computational time for mapping with Yara, Bowtie2 and BWA, respectively. Green and blue bars refer to the two Single Cell samples used for the experiment.

4.2 BioSeqZip on DNA-Seq data

We tested the collapsing procedure provided by BioSeqZip on a 74x coverage DNA-seq paired-end sample (SRR7890958) with the following Library specs:

- Name: FFG_IL_N_6h
- Instrument: Illumina HiSeq 4000
- Strategy: WGS
- Source: GENOMIC
- Selection: RANDOM
- Layout: PAIRED
- Coverage: 74x
- Total reads after trimming: 1,523,752,982

First of all, we tested single-end collapsing running BioSeqZip on the first mate of the sample, experiencing an 18.38% gain in terms of the number of reads in the file which corresponds to 19.65% gain in disk space. Then, we run the BioSeqZip_collapser on the paired-end files experiencing a read compression of 8.70% and a disk space reduction of 10.01%.

The reason of these observations could be that DNA-seq samples, given the origin of the sequenced data, exposes a lower redundancy, leading to lower compression, making the collapsing procedure less effective reducing the gain the user can expect to have in post-collapsing pipelines.

4.3 BioSeqZip - RAM/Runtime trade-off

In Table 1 we explore the trade-off between the maximum amount of RAM BioSeqZip is allowed to use and the number of seconds required for running the collapse procedure.

Table 1: Specification of the raw and collapsed samples part of the BodyMap dataset

	Runtime [s]					
Max RAM	ERR030890		ERR030896		ERR030902	
[GB]	1 thread	4 threads	1 thread	4 threads	1 thread	4 threads
4	377	304	435	379	470	379
8	405	329	467	372	472	397
16	379	336	481	394	512	442
32	301	227	413	300	457	313

The analysis highlights that the runtime required for collapsing a sample slightly increases as the size of the internal buffer, used for storing the sequences, increase. This is due to the algorithmic structure of BioSeqZip: it is composed of an I/O part, which scales linearly with the amount of data to be read or written, and a collapsing part whose core is the buffer sorting engine, which sorts the sequences to be collapsed in alphabetical order. The higher runtime observed when collapsing a larger amount of data is related to the asymptotic complexity of the sorting algorithm being O(n * log(n)). Basically, given X sequences read from the disk in time R and sorted in time S, performing I/O on 2 * X data requires 2 * R, but sorting them requires more than 2 * S.

This trend is broken when the collapser is allowed to use an amount of RAM bigger than the size of the samples (all three samples have approximately 17-18 GB size). In this case, the higher runtime required by the sorting algorithm is balanced by the fact that BioSeqZip does not need to create temporary files on the disk for performing sequences merging, allowing the collapser to used much less I/O operations, which are the real runtime bottleneck in the typical scenario where BioSeqZip operates.

4.4 BioSeqZip+STAR vs Rail-RNA

In table 2 we reports the output of the comparison between samples alignment with Rail-RNA and with the combination of BioSeqZip and STAR. Rail-RNA was run with the default local configuration using 8 running threads, in order to be fair with the previous analysis carried on with STAR. Also, Rail-RNA was not asked to report deliverables different from its default one.

Table 2: Specification of the raw and collapsed samples part of the BodyMap dataset

	Tool	Runtime partials [s]	Runtime total [s]	Runtime total [h]
ERR030888 (single-sample x 75bp)	BioSeqZip collapse	367	2521	0.7
	STAR	1086		
	BioSeqZip expand	1068		
	Rail-RNA default	22892	22892	6.36
BodyMap Single-End (16 samples x 75bp)	BioSeqZip collapse	7763	55047	15.29
	STAR	40255		
	BioSeqZip expand	7029		
	Rail-RNA default	170538	170538	47.37

The exact command line used is:

```
rail-rna go local --m <manifest path>                \
    -x <bowtie index path> <bowtie2 index path>     \
    -p 8 -o <output directory path>
```

As highlighted by the result table the runtime of the pipeline featuring BioSeqZip and STAR is lower than the one of Rail-RNA. However, such comparison is not completely fair: first of all, the information the two alignment tools provide are not the same, with Rail-RNA providing more data than STAR. Then, it should be noticed that Rail-RNA is a cloud-ready alignment tool whose design and implementation may have led to solutions which do not perform best on local and resource-constrained machines, which are the targets BioSeqZip was mainly designed for.

5 Supplementary Tables

Table 3: Specification of the raw and collapsed samples part of the BodyMap dataset

BodyMap 2.0 - Sample statistics								
	RAW		COLLAPSED RECORDS				GAIN	
SAMPLE	RECORDS [M]	SIZE [GB]	RECORDS [M]	SIZE [GB]	TIME [s]	RAM [GB]	RECORDS	SIZE
ERR030872	81.91	24.96	44.24	10.53	655	7.20	45.99%	57.83%
ERR030873	81.84	24.94	43.39	10.33	623	7.20	46.98%	58.60%
ERR030874	80.95	24.67	52.56	12.51	690	7.20	35.06%	49.29%
ERR030875	81.22	24.75	32.01	7.61	585	7.20	60.59%	69.25%
ERR030876	82.11	25.03	30.91	7.35	566	7.20	62.35%	70.62%
ERR030877	82.33	25.09	33.88	8.06	611	7.20	58.85%	67.89%
ERR030878	82.08	25.02	26.77	6.36	567	7.20	67.39%	74.56%
ERR030879	79.30	24.17	24.96	5.93	562	7.20	68.52%	75.45%
ERR030880	77.30	23.56	37.49	8.92	631	7.20	51.50%	62.14%
ERR030881	74.47	22.70	40.94	9.74	583	7.20	45.03%	57.09%
ERR030882	73.51	22.41	53.48	12.73	619	7.21	27.25%	43.19%
ERR030883	75.86	23.12	39.87	9.49	568	7.20	47.44%	58.98%
ERR030884	82.44	25.13	30.07	7.15	590	7.20	63.53%	71.55%
ERR030885	80.40	24.51	32.03	7.62	624	7.20	60.16%	68.92%
ERR030886	82.92	25.28	38.46	9.15	590	7.20	53.61%	63.80%
ERR030887	80.05	24.40	31.40	7.47	569	7.20	60.77%	69.39%
ERR030888	76.27	15.17	22.63	3.70	367	7.93	70.33%	75.62%
ERR030889	76.17	15.15	29.14	4.77	394	7.93	61.74%	68.54%
ERR030890	64.31	12.79	32.68	5.35	337	7.93	49.19%	58.21%
ERR030891	77.20	15.36	26.10	4.27	388	7.93	66.19%	72.21%
ERR030892	80.26	15.97	22.61	3.70	380	7.93	71.83%	76.85%
ERR030893	79.77	15.87	22.93	3.75	379	7.93	71.25%	76.38%
ERR030894	76.77	15.27	27.97	4.58	368	7.93	63.56%	70.05%
ERR030895	77.45	15.41	20.94	3.42	366	7.93	72.96%	77.79%
ERR030896	81.26	16.17	16.84	2.75	401	7.93	79.28%	82.98%
ERR030897	81.92	16.30	19.29	3.15	386	7.93	76.46%	80.66%
ERR030898	83.32	16.58	19.21	3.14	382	7.93	76.95%	81.06%
ERR030899	82.86	16.49	13.68	2.23	350	7.93	83.49%	86.45%
ERR030900	82.79	16.47	20.45	3.34	409	7.93	75.30%	79.70%
ERR030901	81.00	16.12	33.94	5.55	404	7.93	58.10%	65.54%
ERR030902	82.04	16.32	30.38	4.97	409	7.93	62.97%	69.56%
ERR030903	80.25	15.97	28.56	4.67	386	7.93	64.41%	70.74%

Table 4: Runtime report of three experiments: (BWA only) samples alignment using the BWA tool as it is, (BWA + BioSeqZip separate expander) collapsed samples alignment using BWA, considering the runtime of the BioSeqZip-expander tool, (BWA + BioSeqZip embedded expander) collapsed samples alignment using BioSeqZip-BWA, i.e. BWA with the expanding functionalities integrated (BioSeqZip-BWA is available at https://github.com/bioinformatics-polito/BioSeqZip-BWA.git

	BWA only	BWA + BioSeqZip (Separate expander)					BWA + BioSeqZip (Embedded expander)			
Sample	Align	Collapse	Align	Expand	Tot	Gain	Collapse	Align	Tot	Gain
ERR030872	2483	655	1298	1196	3149	-26.82%	655	1234	1889	23.92%
ERR030873	2329	623	1237	1216	3076	-32.07%	623	1192	1815	22.07%
ERR030874	2578	690	1587	1349	3626	-40.65%	690	1520	2210	14.27%
ERR030875	2542	585	1016	1045	2646	-4.09%	585	980	1565	38.43%
ERR030876	2168	566	797	994	2357	-8.72%	566	796	1362	37.18%
ERR030877	2510	611	1029	1042	2682	-6.85%	611	1010	1621	35.42%
ERR030878	2721	567	898	878	2343	13.89%	567	965	1532	43.70%
ERR030879	2414	562	791	920	2273	5.84%	562	761	1323	45.19%
ERR030880	2120	631	1089	1078	2798	-31.98%	631	996	1627	23.25%
ERR030881	2402	583	1358	1106	3047	-26.85%	583	1249	1832	23.73%
ERR030882	1963	619	1493	1319	3431	-74.78%	619	1356	1975	-0.61%
ERR030883	2121	568	1169	1128	2865	-35.08%	568	1070	1638	22.77%
ERR030884	2314	590	903	1002	2495	-7.82%	590	832	1422	38.55%
ERR030885	2254	624	967	1014	2605	-15.57%	624	888	1512	32.92%
ERR030886	1986	590	1001	1133	2724	-37.16%	590	914	1504	24.27%
ERR030887	2131	569	874	1011	2454	-15.16%	569	782	1351	36.60%
ERR030888	1272	367	441	463	1271	0.08%	367	435	802	36.95%
ERR030889	1495	394	656	506	1556	-4.08%	394	642	1036	30.70%
ERR030890	1040	337	602	552	1491	-43.37%	337	585	922	11.35%
ERR030891	1289	388	520	487	1395	-8.22%	388	510	898	30.33%
ERR030892	1299	380	459	471	1310	-0.85%	380	449	829	36.18%
ERR030893	1451	379	488	457	1324	8.75%	379	453	832	42.66%
ERR030894	1215	368	504	507	1379	-13.50%	368	474	842	30.70%
ERR030895	1310	366	403	446	1215	7.25%	366	376	742	43.36%
ERR030896	1468	401	370	436	1207	17.78%	401	350	751	48.84%
ERR030897	1716	386	442	451	1279	25.47%	386	415	801	53.32%
ERR030898	1453	382	407	449	1238	14.80%	382	387	769	47.08%
ERR030899	1248	350	266	434	1050	15.87%	350	251	601	51.84%
ERR030900	1407	409	424	478	1311	6.82%	409	395	804	42.86%
ERR030901	1406	404	690	586	1680	-19.49%	404	645	1049	25.39%
ERR030902	1336	409	599	562	1570	-17.51%	409	560	969	27.47%
ERR030903	1358	386	588	564	1538	-13.25%	386	545	931	31.44%
Paired-End	37036	9633	17507	17431	44571	-20.35%	9633	16545	26178	29.32%
Single-End	21763	6106	7859	7849	21814	-0.23%	6106	7472	13578	37.61%

Table 5: Runtime report of three experiments: (Yara only) samples alignment using the Yara tool as it is, (Yara + BioSeqZip separate expander) collapsed samples alignment using Yara, considering the runtime of the BioSeqZip-expander tool, (Yara + BioSeqZip embedded expander) collapsed samples alignment using BioSeqZip-Yara, i.e. Yara with the expanding functionalities integrated (BioSeqZip-Yara is available at https://github.com/bioinformatics-polito/BioSeqZip-Yara.git

	Yara only	Yara + BioSeqZip (Separate expander)					Yara + BioSeqZip (Embedded expander)			
Sample	Align	Collapse	Align	Expand	Tot	Gain	Collapse	Align	Tot	Gain
ERR030872	1323	655	981	1337	2973	-124.72%	655	1041	1696	-28.19%
ERR030873	1316	623	925	1291	2839	-115.73%	623	860	1483	-12.69%
ERR030874	1442	690	1148	1424	3262	-126.21%	690	1024	1714	-18.86%
ERR030875	1397	585	724	1150	2459	-76.02%	585	668	1253	10.31%
ERR030876	1218	566	566	1021	2153	-76.77%	566	558	1124	7.72%
ERR030877	1419	611	756	1130	2497	-75.97%	611	718	1329	6.34%
ERR030878	1675	567	662	966	2195	-31.04%	567	638	1205	28.06%
ERR030879	1623	562	602	950	2114	-30.25%	562	595	1157	28.71%
ERR030880	1253	631	774	1137	2542	-102.87%	631	720	1351	-7.82%
ERR030881	1713	583	1102	1139	2824	-64.86%	583	1000	1583	7.59%
ERR030882	1367	619	1090	1360	3069	-124.51%	619	981	1600	-17.04%
ERR030883	1258	568	915	1147	2630	-109.06%	568	839	1407	-11.84%
ERR030884	1356	590	707	1045	2342	-72.71%	590	667	1257	7.30%
ERR030885	1550	624	973	1029	2626	-69.42%	624	889	1513	2.39%
ERR030886	1192	590	731	1141	2462	-106.54%	590	688	1278	-7.21%
ERR030887	1222	569	610	1089	2268	-85.60%	569	555	1124	8.02%
ERR030888	597	367	199	521	1087	-82.08%	367	323	690	-15.58%
ERR030889	668	394	302	499	1195	-78.89%	394	349	743	-11.23%
ERR030890	515	337	303	514	1154	-124.08%	337	457	794	-54.17%
ERR030891	590	388	254	474	1116	-89.15%	388	284	672	-13.90%
ERR030892	583	380	208	464	1052	-80.45%	380	301	681	-16.81%
ERR030893	707	379	226	440	1045	-47.81%	379	206	585	17.26%
ERR030894	607	368	252	522	1142	-88.14%	368	232	600	1.15%
ERR030895	676	366	198	487	1051	-55.47%	366	191	557	17.60%
ERR030896	710	401	159	453	1013	-42.68%	401	152	553	22.11%
ERR030897	741	386	183	438	1007	-35.90%	386	177	563	24.02%
ERR030898	726	382	179	462	1023	-40.91%	382	169	551	24.10%
ERR030899	619	350	112	390	852	-37.64%	350	116	466	24.72%
ERR030900	661	409	197	509	1115	-68.68%	409	196	605	8.47%
ERR030901	655	404	325	617	1346	-105.50%	404	315	719	-9.77%
ERR030902	625	409	287	550	1246	-99.36%	409	256	665	-6.40%
ERR030903	710	386	286	525	1197	-68.59%	386	246	632	10.99%
Paired-End	22324	9633	13266	18356	41255	-84.80%	9633	12441	22074	1.12%
Single-End	10390	6106	3670	7865	17641	-69.79%	6106	3970	10076	3.02%
